# A Novel Invertebrate Predator on an Oceanic Island: Impacts and Invasion Dynamics of *Kontikia andersoni* on Macquarie Island

**DOI:** 10.1002/ece3.71663

**Published:** 2025-07-01

**Authors:** Kita M. Williams, Samuel Waite, Melissa Houghton, Jennifer Firn, Justine Shaw

**Affiliations:** ^1^ Queensland University of Technology (QUT) Brisbane Queensland Australia; ^2^ Securing Antarctica's Environmental Future (SAEF) Melbourne Victoria Australia; ^3^ Natural Resources and Environment Tasmania New Town Tasmania Australia; ^4^ Australian Antarctic Division Kingston Tasmania Australia

**Keywords:** biosecurity, ecosystem impact, invertebrates, land planarian, Platyhelminthes, prey preference

## Abstract

Sub‐Antarctic islands, characterised by high endemism and invertebrate‐dominated terrestrial ecosystems, hold significant conservation value and are vulnerable to biological invasion. On Macquarie Island, the invasive terrestrial flatworm *Kontikia andersoni* poses a threat to native biodiversity and ecosystem functioning. To determine the impacts of this invasive predator on native invertebrate communities, we examined species co‐occurrence data and community composition from multiple sites across the island. Our findings indicate that at higher elevations where *K. andersoni* is present, there are significant reductions in invertebrate richness. Additionally, of the abiotic factors tested, slope and topographically deflected wind speed explained the highest amount of variation in flatworm distribution. Habitat suitability modelling identified areas at risk of invasion, providing targets for future biosecurity surveys. This study enhances our understanding of *K. andersoni* invasion dynamics and informs conservation efforts on Macquarie Island.

## Graphical abstract

This study examines the invasion dynamics of the predatory invertebrate flatworm *Kontikia andersoni* , and its impacts on the invertebrate communities of sub‐Antarctic Macquarie Island. Our findings indicate that at higher elevations where *K. andersoni* is present there are significant reductions in invertebrate richness. The abiotic factors slope and topographically deflected wind speed were found to explain the highest amount of variation in flatworm distribution.

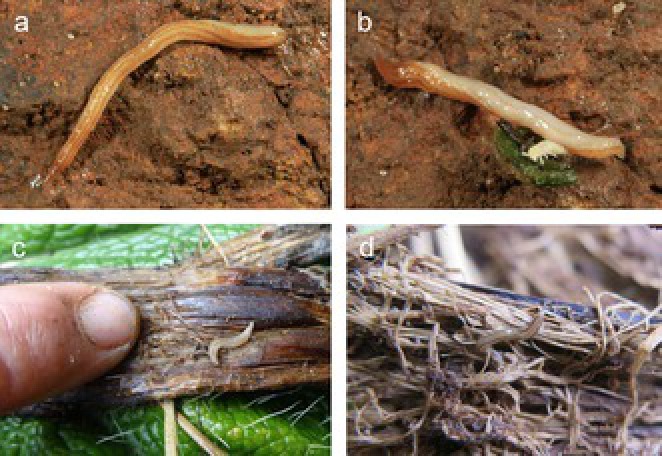

## 1 Introduction

Sub‐Antarctic islands have high conservation value. They are characterised by high endemism and low species richness, with invertebrates dominating native terrestrial fauna (Chown and Convey 2007, 2016; Shaw et al. 2010). Invasive non‐native species are a major threat to biodiversity on oceanic islands, as they can change community structure, alter ecological interactions, displace native taxa, and degrade ecosystem functioning (Géron et al. 2023; Greenslade et al. 2008; Houghton et al. 2019). In the sub‐Antarctic region, the invasion dynamics and impacts of introduced invertebrates are often poorly understood (Lee et al. 2009; Lee and Chown 2016). Some species persist without significantly expanding or contracting their range, whereas only a small number proliferate to become invasive (Frenot et al. 2005; Greenslade 2002; Greenslade et al. 2008; Lebouvier et al. 2020). Identifying the factors that constrain and/or drive these invasions is essential for developing more effective biosecurity and conservation strategies under the unique abiotic and biotic conditions of sub‐Antarctic islands (Chown et al. 2008; Greve et al. 2017; Houghton et al. 2022; Patiño et al. 2017).

**FIGURE 1:**
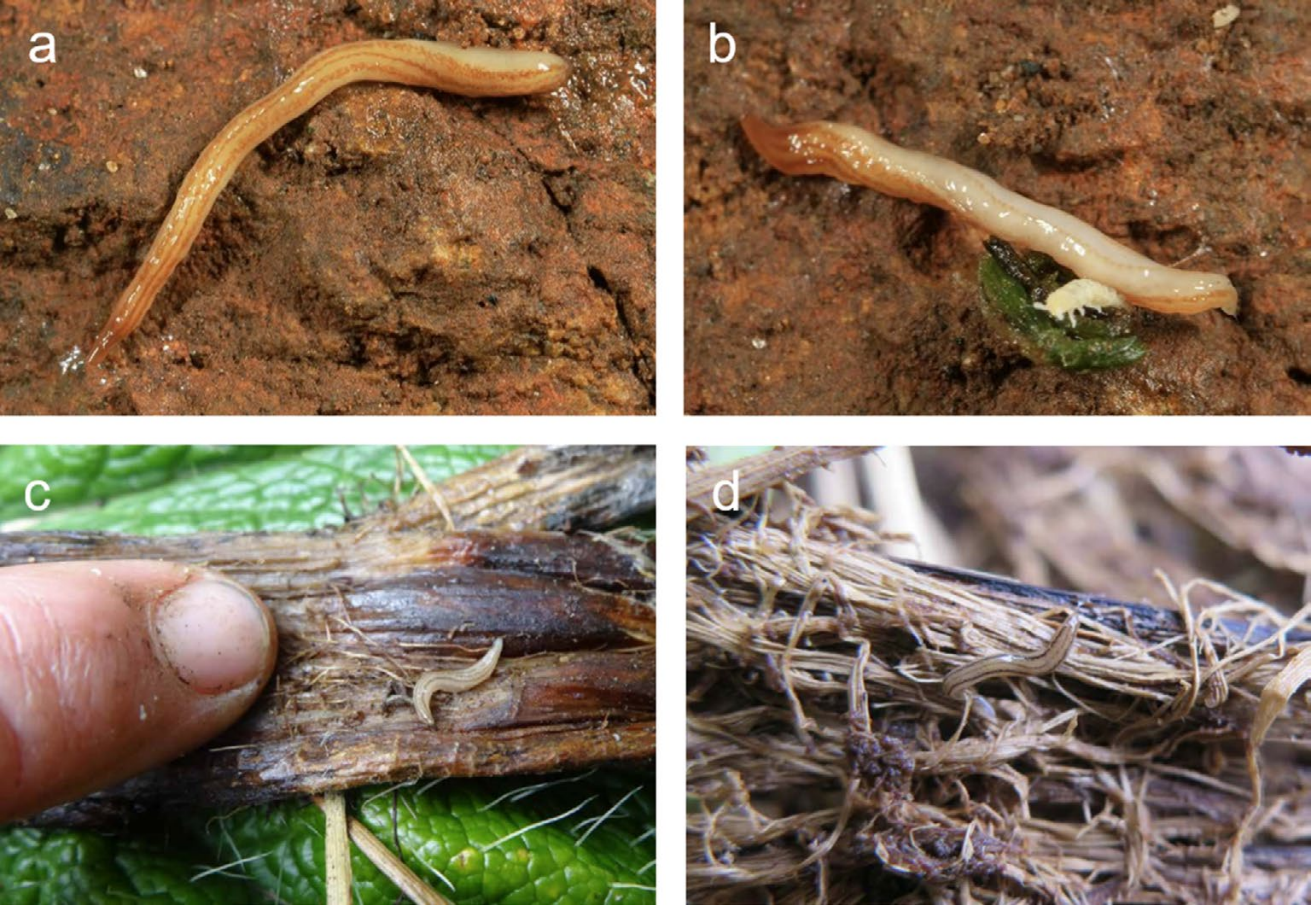
(a) *Kontikia andersoni* in Cornwall, England; (b) *K. andersoni* and a springtail, Cornwall, England; (c, d) *K. andersoni* in *Stilbocarpa* litter and tussock litter, Macquarie Island. Photographs (a, b) by David Fenick; photographs (c, d) by Melissa Houghton.

Macquarie Island, a sub‐Antarctic World Heritage Area situated in the Southern Ocean (54°30′S, 158°57′E), is characterised by tundra‐like vegetation and a cool, wet, and windy climate (Bergstrom et al. 2009; Clements et al. 2007). Non‐native species were transported by humans in the late 1800s (Cumpston 1968). Non‐native vertebrate mammals have subsequently been eradicated (Parks and Wildlife Service 2014; Springer 2016); though several non‐native plants and many non‐native invertebrates persist (Houghton 2020; Whinam et al. 2014). Among these are two non‐native terrestrial flatworms (also known as land planarians), *Kontikia andersoni* (Figure 1) and *Arthurdendys vegrandis* (Platyhelminthes: Geoplanidae), thought to be introduced from New Zealand during commercial sealing and penguin harvesting operations in the 19th century (Greenslade et al. 2007; Winsor and Stevens 2005). *Kontikia andersoni* has since expanded its range, spreading ~500 m/year and reaching elevations of ~170 m, far exceeding original predictions (Houghton et al. 2022). *Arthurdendys vegrandis* has not been detected in high numbers in recent years (Houghton et al. 2022), and as such is not the focus of this study.

Terrestrial flatworms are significant predators in ecosystems where they have been introduced, severely impacting native invertebrate populations and disrupting nutrient cycling (Boll and Leal‐Zanchet 2016; Jones and Boag 1996; Wardle et al. 2009). Knowledge of terrestrial flatworm prey preference and predation frequency is limited for many species (including *K. andersoni* ); however, many flatworms are generalist and opportunistic predators, capable of adapting their diet to local prey, facilitating their spread across new regions (Boll et al. 2015; Justine et al. 2014). Prey invertebrates can include snails and slugs, earthworms, isopods, spiders and harvestmen, insects and insect larvae, springtails, mites, and other flatworms (Boll et al. 2015; Cseh et al. 2017; Nakamori and Suzuki 2012). Flatworms are capable of preying on very small invertebrates (Iwai et al. 2010) and can also feed on larger prey through gregarious attacks involving multiple individuals (Boll et al. 2015; Ogren 1995; Sugiura 2010a; Winsor and Stevens 2005). Flatworms may also scavenge dead invertebrates (Boll et al. 2015; Ohbayashi et al. 2005).

On Macquarie Island, increasing elevation and associated environmental conditions, e.g.,—lower temperatures, higher wind speeds, and reduced prey availability,—are likely to influence habitat suitability and the distribution of *K. andersoni* , as has been found for other invertebrates in sub‐Antarctic environments (Chown and Convey 2016; Davies and Melbourne 1999; Ouisse et al. 2020; Terauds et al. 2011). Moisture is vital for flatworms, as they are sensitive to desiccation (Froehlich 1955; Gerlach 2019; Sugiura 2009). It has also been suggested that flatworm proximity to walking tracks increases the probability of human‐mediated dispersal (Greenslade et al. 2007; Houghton et al. 2022). Additionally, recovering vegetation following the eradication of non‐native rabbits in 2014 may have expanded suitable habitat for the flatworm (Fitzgerald et al. 2021). As a predator capable of significant ecological disruption, *K. andersoni* poses a threat to the unique invertebrate‐dominated ecosystems of Macquarie Island. Understanding the ecological impacts, habitat preferences, and distribution of the flatworm is critical to informing targeted conservation actions and ensuring the long‐term protection of this World Heritage Area.

Here, we investigate the invasive dynamics and ecological impacts of the flatworm *K. andersoni* on Macquarie Island. Specifically, we aim to: (1) assess the impact of *K. andersoni* on native and non‐native invertebrate communities, by identifying potential prey and evaluating predation pressure at sites where the flatworm is present; (2) examine differences in invertebrate community composition between sites with and without *K. andersoni* ; and, (3) identify environmental variables influencing flatworm distribution and model suitable habitat on the island. We hypothesise that *K. andersoni* is capable of preying on a wide range of invertebrates, increasing predation pressure in areas where it is present, and that its distribution is influenced by elevation, topographic wetness, and proximity to walking tracks. This research will inform future targeted monitoring, and effective biosecurity and conservation actions on Macquarie Island, particularly in habitats identified as high‐risk.

## 2 Methods

### 2.1 Flatworm Distribution

We used data from a 2015 to 2018 island‐wide invertebrate trapping program (doi:10.26179/5ba86d73e923c), and dedicated flatworm survey (Houghton et al. 2022). The flatworm survey conducted in 2018 noted the presence of flatworms at 29 of the 103 sites island‐wide. Refer to Houghton et al. (2022) for survey methodology.

### 2.2 Identifying Flatworm Prey

Data from a 2015 to 2018 invertebrate trapping survey (doi:10.26179/5ba86d73e923c) across 24 sites (Appendix A, Table A1) were examined to determine which invertebrate species co‐occur with *K. andersoni* on Macquarie Island. In this survey, invertebrates had been identified to species level where possible. Acarina, Annelida and Collembola were classified to Class/Order only due to the high diversity or high number of individuals (e.g., Collembola commonly reach densities of 30,000 m^−2^ (Greenslade and Van Klinken 2006)). A literature search was undertaken to investigate the flatworm prey identified from other regions. These compiled data are listed in the Appendix A, Table A2. The invertebrates found at each site on Macquarie Island were each assigned to trophic groups (predator/parasitoid, herbivore, detritivore/omnivore) based on literature (Greenslade and Van Klinken 2006; Houghton 2020). A conceptual interaction network was created for the sites with and without *K. andersoni* present.

### 2.3 Ecological Impacts

All statistical analyses were conducted in RStudio (RStudio Team 2023) using the R programming language (R Core Team 2023). Invertebrate abundance data from the 2015 to 2018 invertebrate pitfall trapping program was collated and averaged for each of the 24 sites. Species with an abundance lower than 5 across all sites and all years were considered rare or transient and removed. Differences in invertebrate community composition were visualised using Non‐metric Multi‐Dimensional Scaling (NMDS) with 999 separate runs of the Bray similarity Matrix, using the *vegan* package (Oksanen et al. 2019). Significance level *α* = 0.05.

Invertebrate communities at 12 sites were investigated to assess differences according to vegetation, elevation, and the presence/absence of *K. andersoni* . We conducted a PERmutational Multivariate Analysis of Variance (PERMANOVA), using the *adonis2* function in the *vegan* package, with 999 permutations for the Bray similarity matrix (Oksanen et al. 2019). Species abundances were square root transformed prior to analysis to adjust for dominant taxa.

### 2.4 Habitat Suitability Modelling

We utilised spatial and environmental data (Bricher et al. 2013; Harris 2015a, 2015b) to model the abiotic and biotic factors influencing the distribution of *K. andersoni* , and to predict future spread into suitable habitats. Eight variables with available island‐wide spatial data were initially chosen (see Appendix A, Table A3 for details of variable ecological significance). Variables were grouped into two categories: (1) biotic disturbance that is, proximity to walking tracks, seal or penguin colonies, and watercourses or lakes; and (2) terrain/topoclimatic that is, aspect, elevation, slope, topographic wetness index, topographically deflected wind speed. All three proximity raster layers had a 5 m^2^ resolution and were created from polygon vector layers sourced from the Australian Antarctic Data Centre (Harris 2015a, 2015b) using SAGA 7.2 (Conrad et al. 2015) with the Saga NextGen Provider plugin in Quantum GIS (QGIS Development Team 2023). Terrain variables raster data were derived from a five‐metre resolution DEM of Macquarie Island created from Airborne Synthetic Aperture RADAR data acquired in 2000 (Bricher et al. 2013). Layer values were extracted for each site surveyed using the Point Sampling Tool plugin (QGIS Development Team 2023).

Pairwise correlations between all continuous variables were assessed and a threshold of 0.7 (Pearson correlation coefficient) was used to determine variable independence (Fitzgerald et al. 2022; Green 1979). Elevation was omitted from modelling due to high correlation with proximity to seal or penguin colonies (pairwise correlation coefficient = 0.8). The remaining variables (seven in total) were retained for modelling (Appendix A, Table A3).

Generalised Additive Models (GAMs) (Hastie and Tibshirani 1986) via the *mgcv* package (Wood 2017) were utilised to model the environmental factors influencing *K. andersoni* distribution. A binomial distribution was specified, and the model smoothing parameter was estimated via Restricted Maximum Likelihood (REML). Model term selection was performed automatically via the ‘shrinkage’ approach (Marra and Wood 2011), with non‐significant variables dropped and the model refitted using the remaining terms (see Tables S1–S6 for further details).

A five‐model suite approach, consisting of GAMs (Hastie and Tibshirani 1986), Boosted Regression Trees (BRTs) (Elith et al. 2008), Random Forests (RF) (Breiman 2001), Artificial Neural Networks (ANNs) (Lek and Guégan 1999) and Extreme Gradient Boosting (XGBoost) (Chen and Guestrin 2016), was trialled to predict *K. andersoni* occupancy and habitat suitability at an island‐wide scale. Model training, testing and hyperparameter tuning was conducted through the *caret* (Kuhn 2008) using the following packages: *mgcv* (GAMs), *gbm* (Greenwell et al. 2022) (BRTs), *ranger* (Wright and Ziegler 2017) (RFs), *nnet* (Venables and Ripley 2002) (ANNs) and *xgbDART* (Rashmi and Gilad‐Bachrach 2015) (XGBoost).

To combat overfitting, models with a mean accuracy of < 0.4 (BRT and XGBoost) were retrained and tuned without their two least important predictors (topographic wetness index and proximity to walking tracks for both). The best‐performing model of the two was subsequently trained on the entire training data set and evaluated on the test set using the Cohen Kappa Statistic (Cohen 1960). If model performance was > 0.4, it was then used to predict the probability of *K. andersoni* occurrence at a 5 m resolution across Macquarie Island.

## 3 Results

### 3.1 Identifying Flatworm Prey

*Kontikia andersoni* was found to occur at six of the 24 invertebrate survey sites (see Houghton et al. 2022). At these sites, we detected up to 31 invertebrate taxa co‐occurring with *K. andersoni* , with 27, 15, 25, 22, 24, and 21 taxa at sites Green Gorge, Rockhopper Bay escarpment edge, Hurd Point, Waterfall Bay, Sandell Bay, and Lusitania Bay respectively (Table 1). For trophic groupings, the most speciose group was detritivores/omnivores (17), followed by equal numbers of herbivores and predators/parasitoids (8). The native predators were spiders (3 species), beetles (3 species), and a parasitic wasp (1 species), along with predatory Acarina (not identified to species level).

**TABLE 1:** Invertebrates found to co‐occur with *K. andersoni* across six sites on Macquarie Island, 2015–18. Functional groups include predators and parasitoids (red), herbivores (green), and detritivores/omnivores (yellow).

Functional group		Phylum/order	Family	Species	Number and name of co‐occurrence sites						
					#	GG	RB	HP	WB	SB	LB
	Omnivores/	Acarina			6	X	X	X	X	X	X
	Predators										
	Detritivores	Annelida			6	X	X	X	X	X	X
	Detritivores	Collembola			6	X	X	X	X	X	X
	Predator	Araneae	Linyphiidae	*Haplinis mundenia*	6	X	X	X	X	X	X
	Predator	Coleoptera	Staphylinidae	*Leptusa antarctica*	6	X	X	X	X	X	X
	Detritivore	Diptera	Australimyzidae	*Australimyza macquariensis*	6	X	X	X	X	X	X
	Detritivore	Diptera	Dolichopidae	*Thinophilus* (*Schoenophilus*) *pedestris pedestris*	6	X	X	X	X	X	X
	Herbivore	Mollusca	Agriolimacidae	*Derocerus reticulatum* †	6	X	X	X	X	X	X
	Herbivore	Mollusca	Punctidae	*Phrixgnathus hamiltoni*	6	X	X	X	X	X	X
	Herbivore	Psocoptera	Pseudocaeciliidae	*Austropsocus insularis*	6	X	X	X	X	X	X
	Herbivore	Thysanoptera	Thripidae	*Physemothrips chrysodermus*	6	X	X	X	X	X	X
	Predator	Araneae	Linyphiidae	*Parafroneta marrineri*	5	X		X	X	X	X
	Detritivore	Diptera	Psychodidae	*Psychoda* sp.	5	X		X	X	X	X
	Detritivore	Diptera	Psychodidae	*Psychoda surcoufi*	5	X		X	X	X	X
	Detritivore	Copepoda	Harpacticoida		5	X	X	X	X		X
	Detritivore	Nematoda			5		X	X	X	X	X
	Predator	Araneae	Desidae	*Myro kerguelensis*	4	X	X		X	X	
	Predator	Coleoptera	Staphylinidae	*Stenomalium sulcithorax*	4	X		X	X		X
	Detritivore	Diptera	Coelopidae		4	X		X	X	X	
	Detritivore	Diptera	Psychodidae	*Psychoda alternata*	4	X		X	X	X	
	Detritivore	Diptera	Tipulidae	*Trimicra pilipes*	4	X		X	X	X	
	Herbivore	Hemiptera	Aphididae	*Jacksonia papillata* †	4	X	X			X	X
	Predator	Coleoptera	Staphylinidae	*Omaliinae* sp.	3	X		X			X
	Detritivore	Diptera	Calliphoridae	*Xenocalliphora* *flavipes* †	3	X		X			X
	Predator/	Hymenoptera	Diapriidae	*Spilomicrus latigaster*	3			X		X	X
	Parasitoid										
	Herbivore	Lepidoptera	Pyralidae	*Eudonia mawsonii*	3	X		X		X	
	Detritivore	Diptera	Chironomidae	*Smittia* sp.	2	X				X	
	Herbivore/	Tardigrada			2			X	X		
	Omnivore										
	Detritivore	Diptera	Ephydrididae	*Ephydrella macquariensis*	1					X	
	Detritivore	Diptera	Sciaridae	*Bradysia strenua* †	1	X					
	Herbivore	Hemiptera	Aphididae	*Myzus ascalonicus* †	1	X					

*Note:* Site names are abbreviated to GG (Green Gorge), RB (Rockhopper Bay Escarpment Edge), HP (Hurd Point), WB (Waterfall Bay), SB (Sandell Bay), and LB (Lusitania Bay). Non‐native species indicated with †.

At all sites, *K. andersoni* co‐occurred with Acarina (mites), Annelida (worms), and Collembola (springtails); these groups were not identified to species level due to their exceptionally high abundance. There are at least 119 species of Acarina (43 families), 15 species of Annelida in the class Oligochaeta, and 34 species of Collembola known from Macquarie Island (Greenslade and Van Klinken 2006; Phillips et al. 2017). Macquarie Island meiofauna include 26 taxa within Nematoda (many undescribed) and 28 species within Tardigrada (Greenslade and Van Klinken 2006). The following species co‐occurred with *K. andersoni* across all sites: spider *Haplinis mundenia* , rove beetle *Leptusa antarctica*, flies *Australimyza macquariensis* and *Thinophilus (Schoenophilus) pedestris pedestris*, introduced slug *Derocerus reticulatum*, snail *Phrixgnathus hamiltoni*, book louse *Austropsocus insularis*, and thrips *Pysemothrips chrysodermus*. Taxa that were less common and co‐occurred with *K. andersoni* at less than three sites were flies *Smittia* sp., Tardigrada, the flies *Ephydrella macquariensis* and *Bradysia strenua*, and the aphid *Myzus ascalonicus*.

**FIGURE 2:**
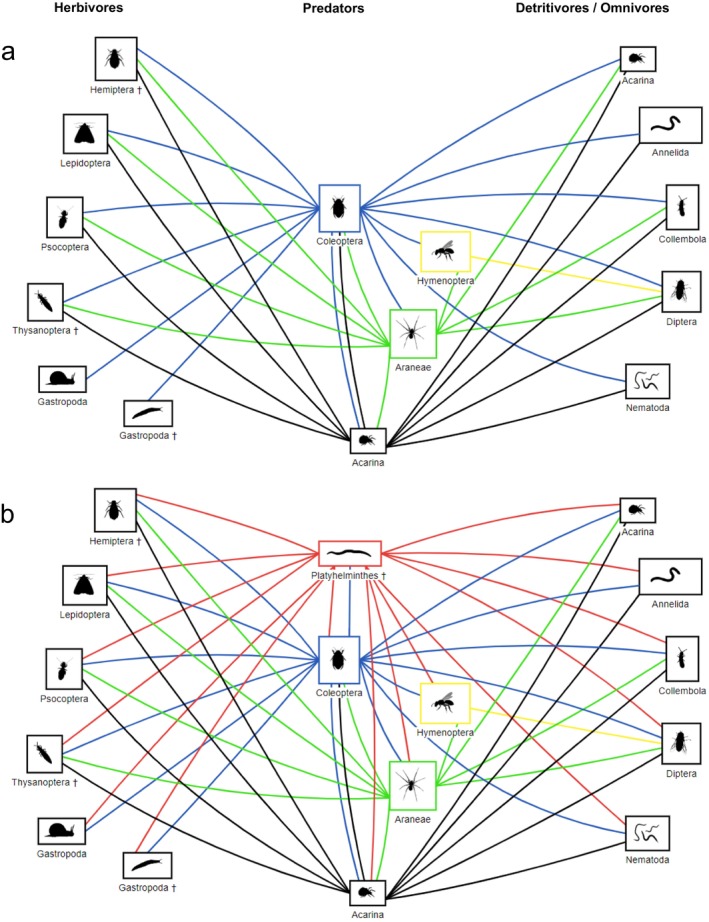
(a) Conceptual interaction network at sites with *K. andersoni* absent, and (b) at sites with *K. andersoni* present. Predation indicated by lines: Acarina (black), Araneae (green), Coleoptera (blue), Hymenoptera (yellow), and Platyhelminthes (red). Non‐native invertebrate taxa indicated with †.

Figure 2a shows a conceptual diagram of native invertebrate communities on Macquarie Island. Species, or groups have been assigned to trophic guilds based on the literature. Figure 2b indicates how the introduction of *K. andersoni* into the community alters community structure and species interactions through predation. This interaction network was constructed based on prey and dietary studies of flatworms (Appendix A, Table A2). We deemed all co‐occurring invertebrates as potential prey of *K. andersoni* , apart from Copepoda and Tardigrada, due to their very small body size. Figure 2b shows how *K. andersoni* increases the predation pressure within invertebrate communities, and competes with native predators for invertebrate preys. Predation could also mutually occur between *K. andersoni* and the native Staphylinidae (Gibson et al. 1997).

**FIGURE 3:**
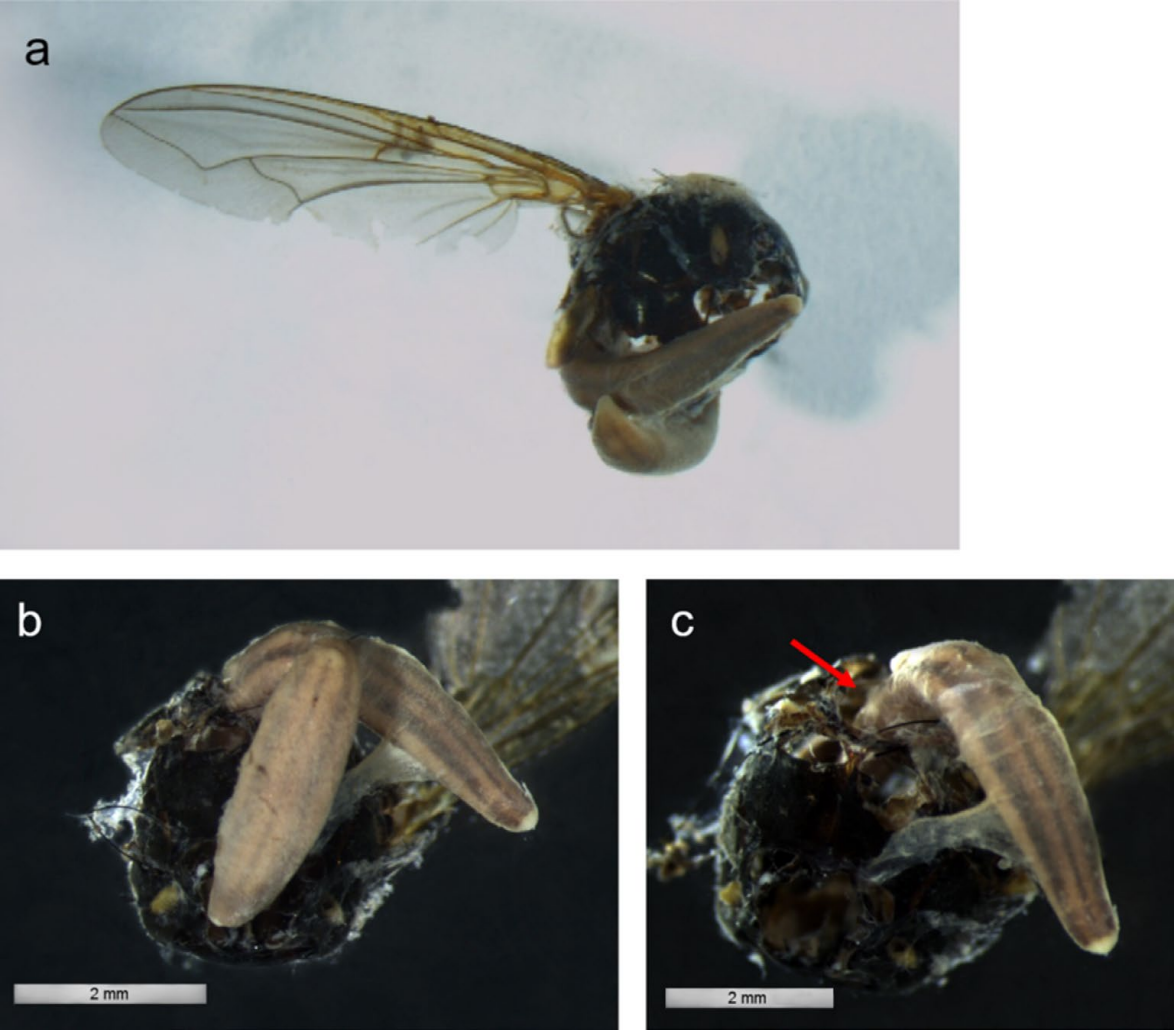
(a, b) Preserved specimen of two *K. andersoni* individuals feeding on the decayed thorax of a *X.* *flavipes*. blowfly, collected from a pitfall trap on Macquarie Island in 2018 by Melissa Houghton. (c) Specimen with the smaller *K. andersoni* removed; the larger flatworm can be seen partially inside the blowfly thorax (red arrow). Photographs by Kita Wiliams.

A pitfall trap collected in 2018 from Green Gorge included two flatworms feeding on a decaying blowfly (*Xenocalliphora* *flavipes*) (Figure 3). This indicates that *K. andersoni* will also scavenge dead invertebrates, and therefore compete with detritivores.

### 3.2 Ecological Impacts

**FIGURE 4:**
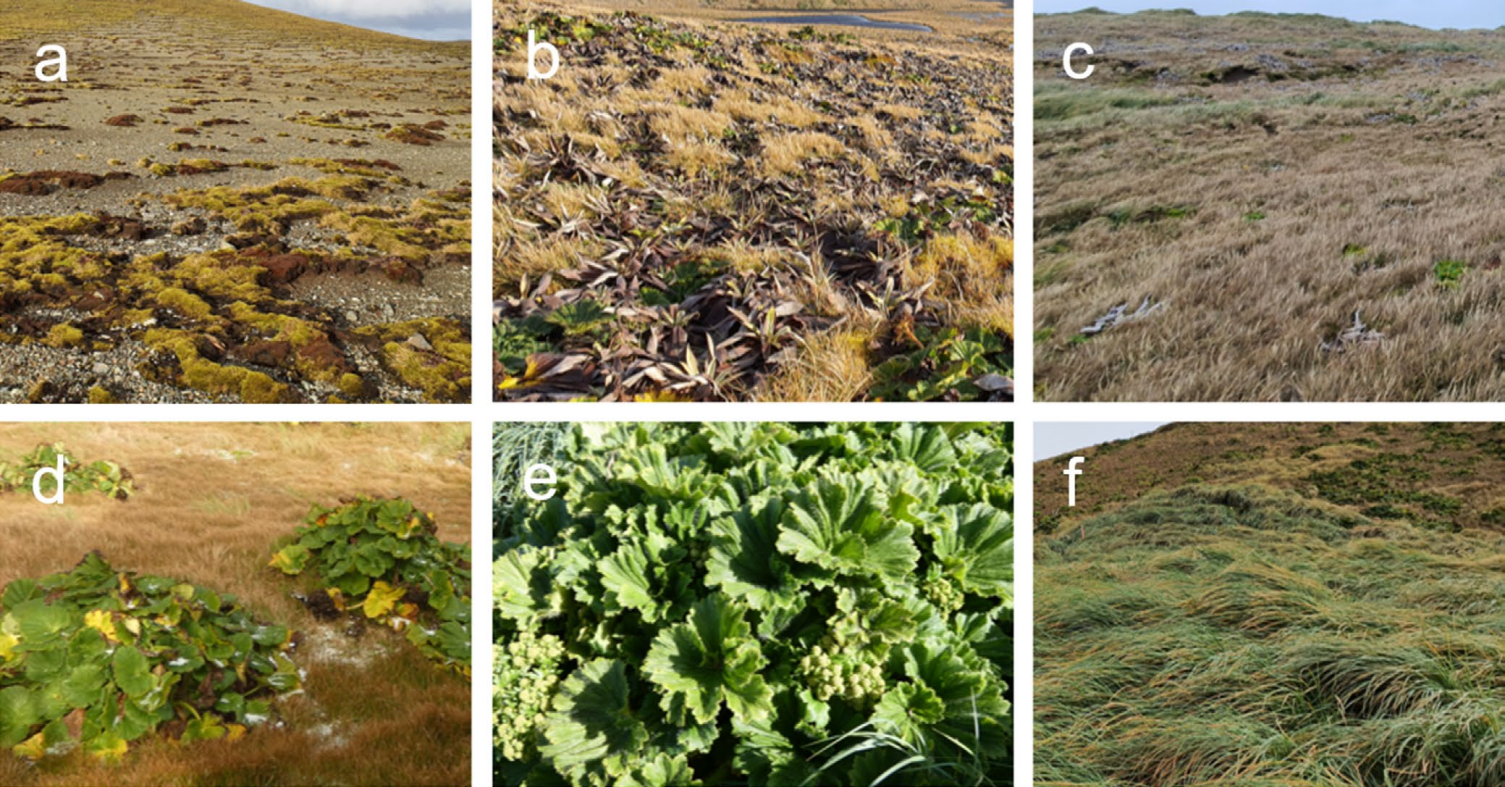
Vegetation types on Macquarie Island: (a) Feldmark, (b) Herbfield, (c) Shortgrass, (d, e) *Stilbocarpa polaris* (Macquarie Island cabbage), (f) Tallgrass. Photographs by Kita Williams and Justine Shaw.

**FIGURE 5:**
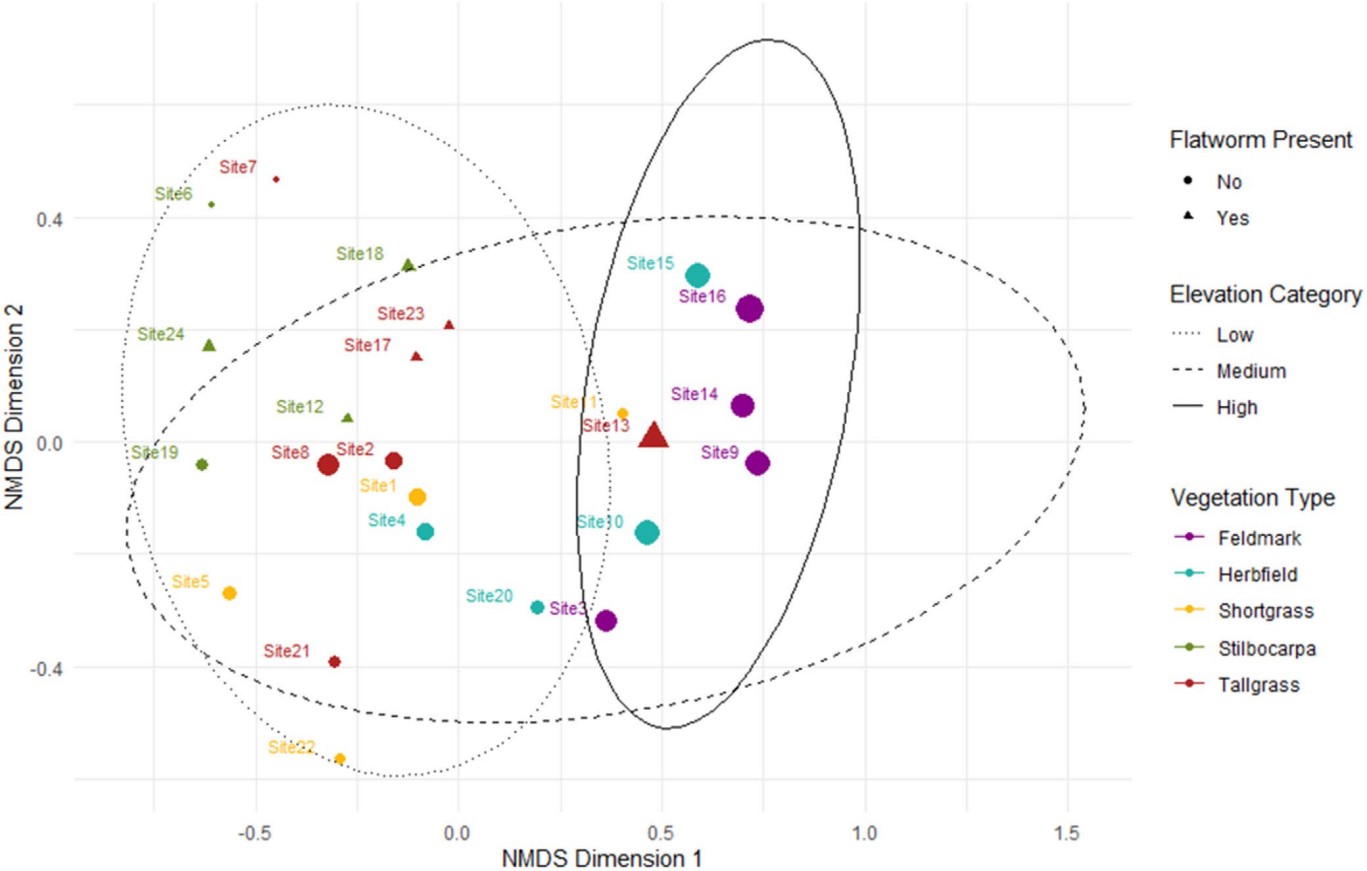
NMDS ordination plot for invertebrate communities within different vegetation types (colours), and with presence/absence of *K. andersoni* (shapes). Elevation is indicated for each site (size), and with ellipses for elevation category (Low 0–100 m, Medium 100–200 m, High 200–300 m). NDMS stress = 0.142.

The 24 surveyed sites spanned five different vegetation types (Figure 4). The NMDS ordination analysis (Figure 5) revealed dissimilarities in invertebrate communities according to vegetation type, elevation, and *K. andersoni* presence/absence.

**FIGURE 6:**
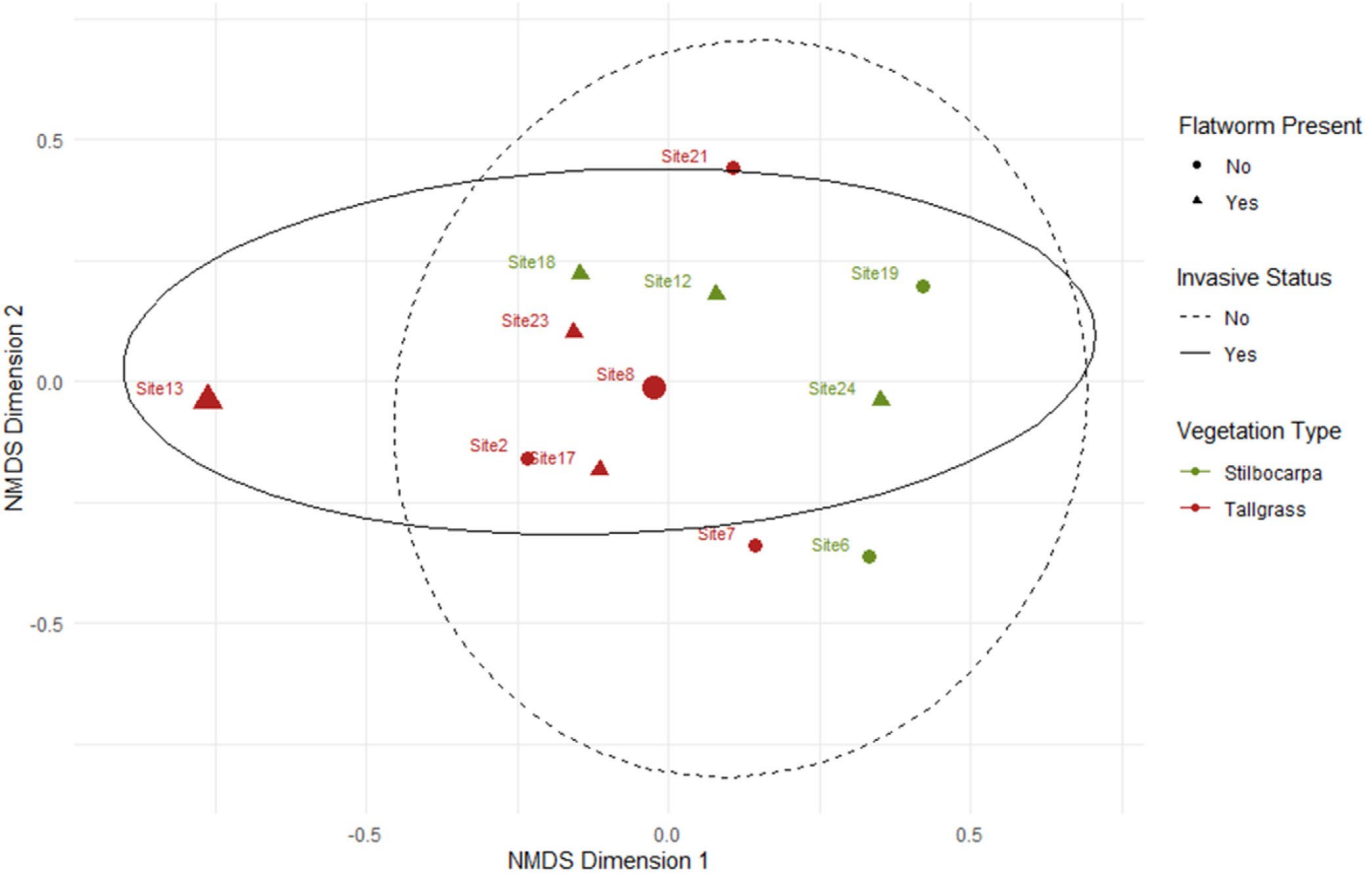
NMDS ordination plot for invertebrate communities within *Stilbocarpa* and Tallgrass (colours) across different elevations (size), and with the presence/absence of *K. andersoni* (shapes and ellipses). NDMS stress = 0.166.

Twelve sites were selected for further analysis. *Kontikia andersoni* was only present in either tall tussock grass (*Poa foliosa*) or *Stilbocarpa* herbfield (*Stilbocarpa polaris*) vegetation. Sites with different vegetation (Feldmark, Herbfield, Shortgrass) were therefore excluded from PERMANOVA analysis. Six sites with *K. andersoni* present were Tallgrass (*n* = 3) and *Stilbocarpa* (*n* = 3). Sites with *K. andersoni* absent were Tallgrass (*n* = 4) and *Stilbocarpa* (*n* = 2). PERMANOVA revealed significant differences in invertebrate communities according to elevation (*R* ^2^ = 0.194, *F* = 2.897, *p* = 0.019), and between the presence and absence of *K. andersoni* (*R* ^2^ = 0.171, *F* = 2.556, *p* = 0.025). The Tallgrass and *Stilbocarpa* communities were not significantly different. The interaction of vegetation type x *K. andersoni* was not significant, indicating that the effect of *K. andersoni* did not differ between the two vegetation types. Site 13 (Rockhopper Bay Escarpment Edge) was found to be the most dissimilar to other sites (Figure 6).

### 3.3 Distribution Parameters

**FIGURE 7:**
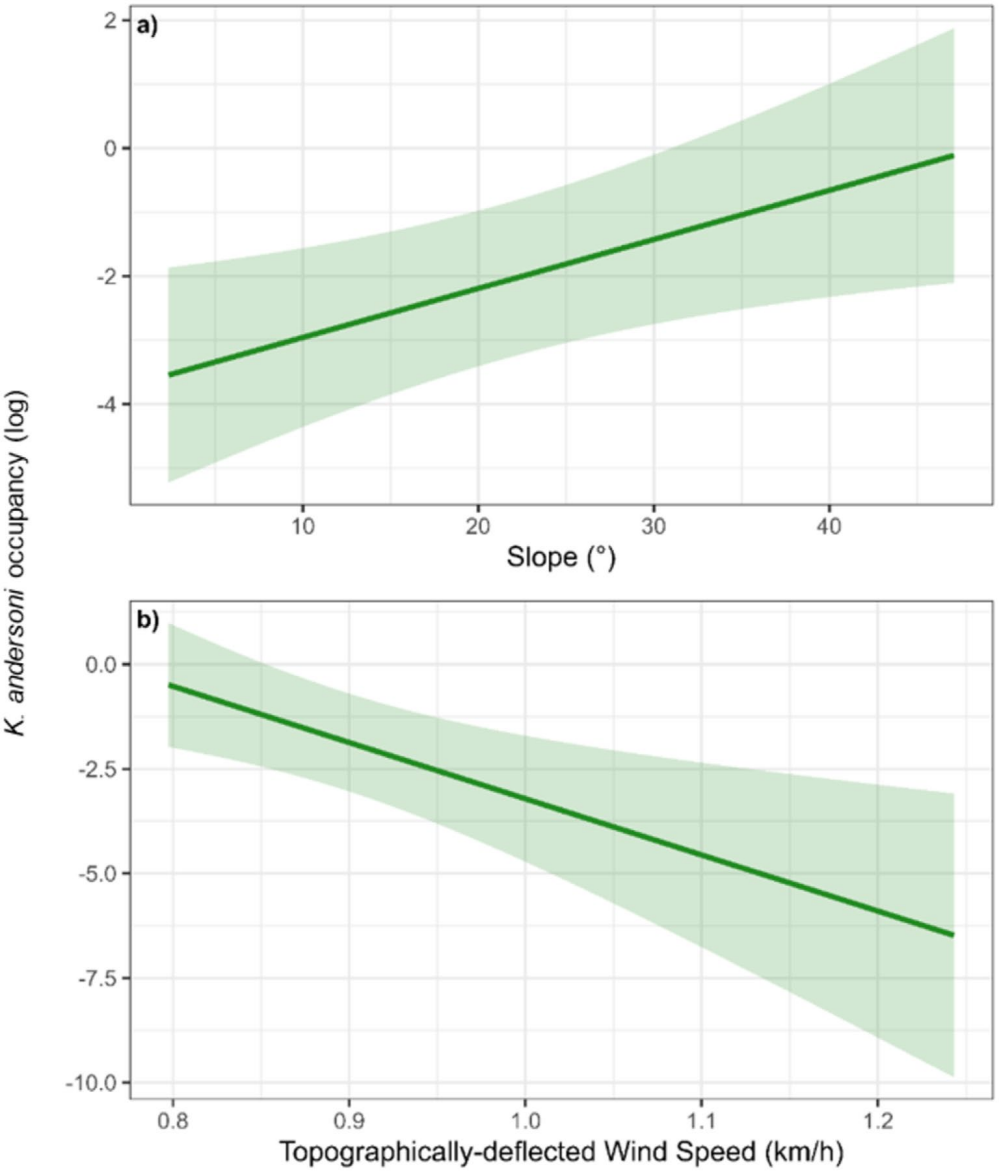
Partial effects plots of the environmental parameters included in the refitted model of *K. andersoni* occupancy: (a) Slope (°) and (b) Topographically deflected wind speed (km/h). Shaded regions represent the 95% confidence interval.

Slope and topographically deflected wind speed were found to be significantly correlated with *K. andersoni* occurrence within the refitted model (Table 2). A linear increase in the odds of *K. andersoni* presence was observed with an increase in slope angle, with the odds of *K. andersoni* occurrence increasing by a factor of ~39.0 from sites with a slope of ~2° to ~47° (Figure 7a). In contrast, the odds of *K. andersoni* occurrence decreased linearly with an increase in site wind speed. From sites with a mean topographically deflected wind speed of 0.8–1.24 km/h, the odds of *K. andersoni* occupancy decreased multiplicatively by a factor of ~47/50000 (Figure 7b). As expected, mean effect uncertainty decreased with a reduction in site observations for both parameters, particularly at sites above 1.1 km/h and 30°.

**TABLE 2:** Refitted generalised additive model statistics for *K. andersoni* . Effective degrees of freedom (EDF), the chi‐squared statistic (Chi. Sq) and *p*‐value are reported.

Explanatory variables	EDF	Chi. sq	*p*
Aspect	—	—	—
Slope	1	6.02	**0.1**
Wetness	—	—	—
Wind	1	8.20	**0.004**
Proximity to penguin or seal colonies			
Proximity to walking tracks	—	—	—
Proximity to water courses			
Easting and northing	6.30	15.1	0.08
Deviance explained (%)	46.5		

*Note:* Significant (*p*‐value < 0.05) variables are highlighted in **bold** and ‘–’represent no data.

### 3.4 Habitat Suitability Modelling

**FIGURE 8:**
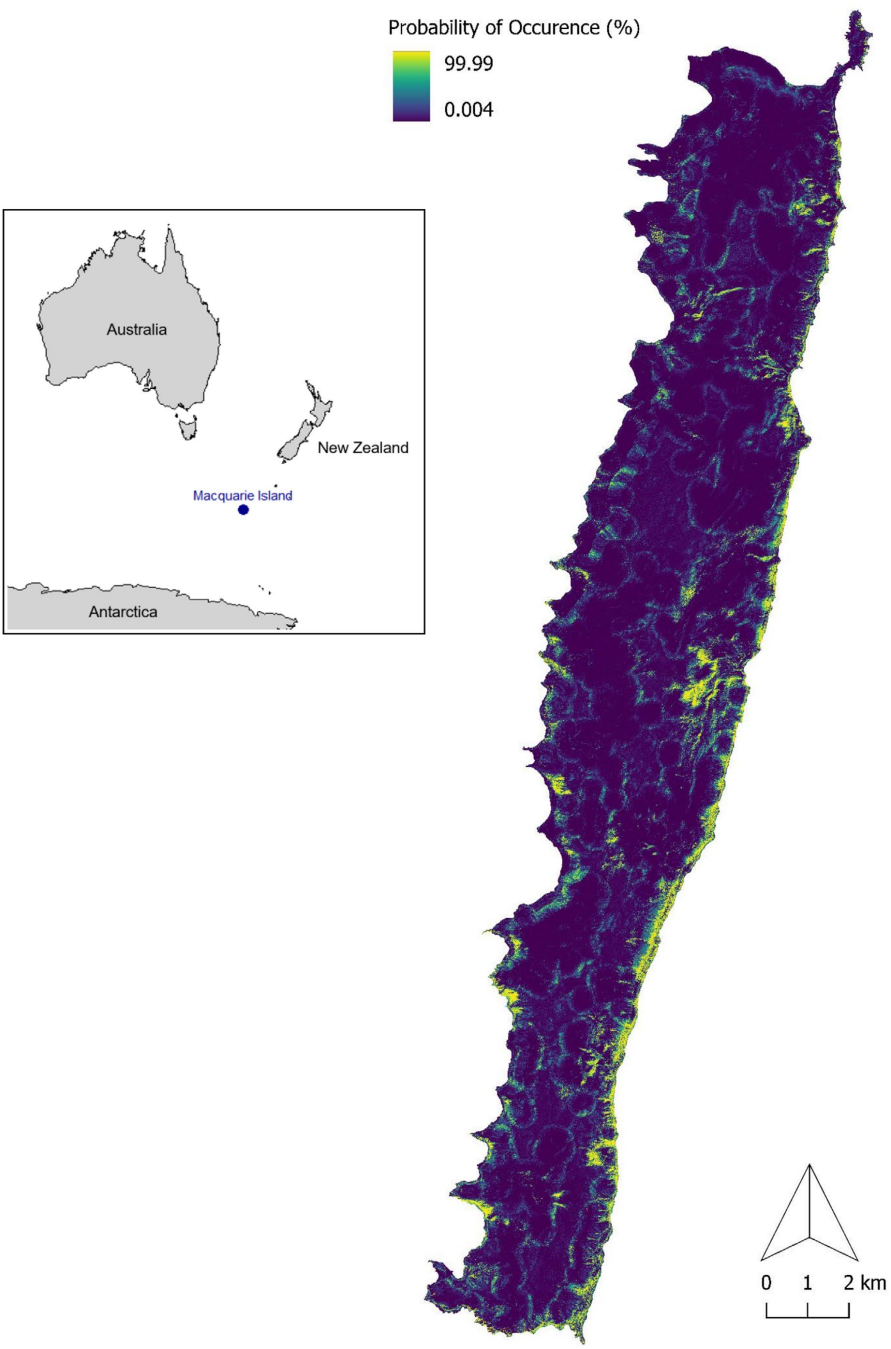
Sub‐Antarctic Macquarie Island (inset). Predicted occurrence probability (%) for *K. andersoni* at a 5 m spatial resolution on Macquarie Island.

**FIGURE 9:**
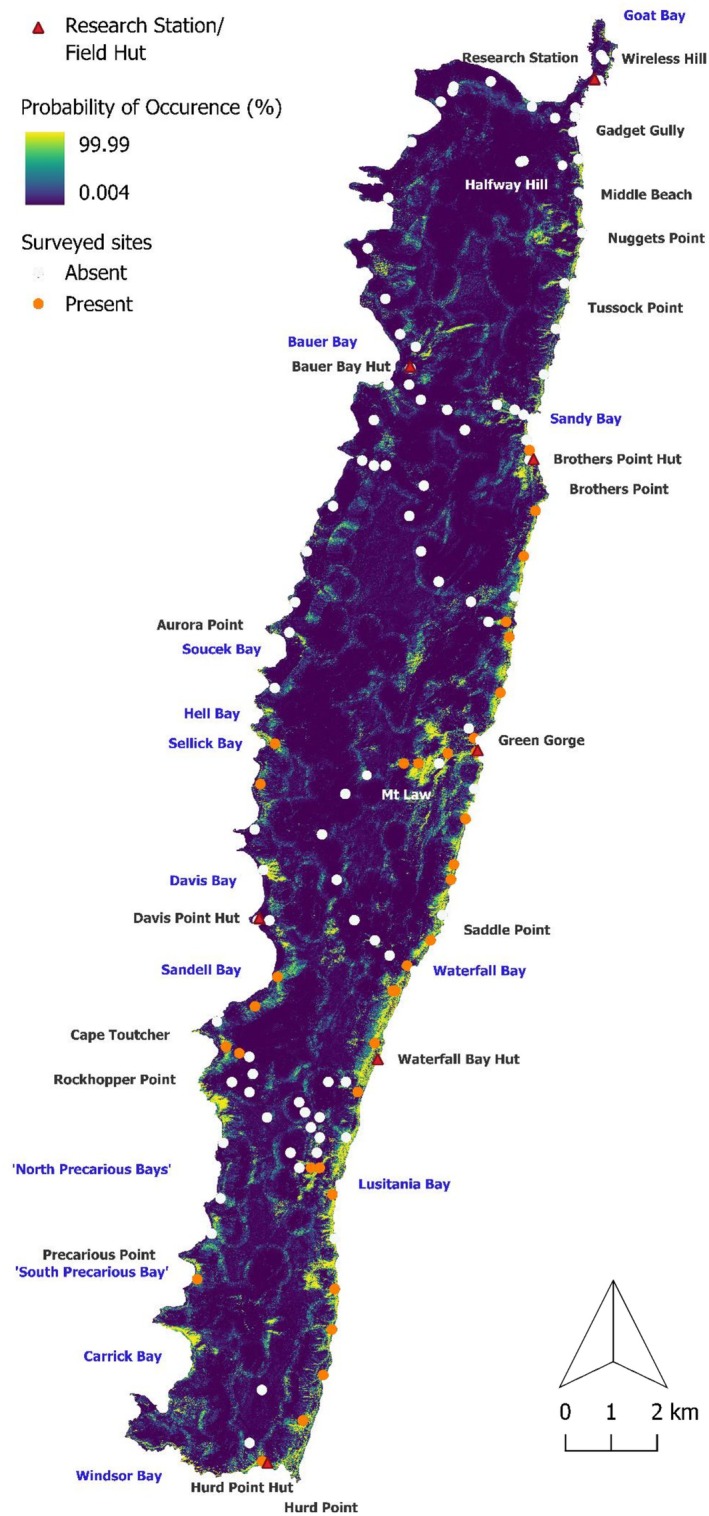
Predicted occurrence probability (%) for *K. andersoni* at a 5 m spatial resolution on Macquarie Island with flatworm surveyed sites overlaid. Sites are categorised into those with *K. andersoni* present (orange dots) and absent (white dots).

Our models indicate several areas with suitable habitats where flatworms have not yet been detected. These include the coastline between Sandy Bay and Wireless Hill, areas inland from Tussock Point and Nuggets Point, scattered zones throughout the middle of the island plateau, and several areas in the middle and southwest coasts of the island, including Davis Bay, Hell Bay, and Aurora Point (Figures 8 and 9).

Models point to additional regions around Mt. Law, Green Gorge, Waterfall Bay and Lusitania Bay where *K. andersoni* is likely to be detected, due to the proximity with current habitats. Suitable habitats exist that have not yet been surveyed, such as the coastal regions around Rockhopper Point and Carrick Bay, and some areas west and east of Hurd Point Hut.

## 4 Discussion

### 4.1 Prey and Ecological Impacts

Flatworms are often generalist predators of insects and their larvae (Cseh et al. 2017; Cuevas‐Caballé et al. 2019). We identified 20 native and five non‐native species co‐occurring with *K. andersoni* as potential prey. Taxa not identified to species level (Acarina, Annelida, Collembola, and Nematoda) were also found to be suitable prey (Appendix A, Table A2). Concerningly, *K. andersoni* is likely to predate endemic species on Macquarie Island, including the snail *Phrixgnathus hamiltoni*, larvae of the moth *Eudonia mawsonii*, and several Collembolans. Populations of native earthworms, spiders, and insect larvae (including many Diptera) may also be heavily impacted, as these taxa are commonly predated by flatworms (Appendix A, Table A2). There is no evidence in the literature of flatworms predating booklice (Psocoptera), aphids (Hemiptera), thrips (Thysanoptera), or wasps (Hymenoptera). However, as flatworms can adapt their diet to include novel invertebrates (Boll et al. 2015; Justine et al. 2014), in the context of Macquarie Island, we consider these taxa to be potential prey. Due to the high density of nematodes in vegetation and soils, and their size of 0.5–3 mm (Greenslade and Van Klinken 2006), nematodes are also considered to be viable prey for *K. andersoni* .

The flatworm may prey upon non‐native invertebrates, including the slug *D. reticulatum* and larvae of the introduced flies *X. flavipes* and *B. strenua* . Flatworms will often predate isopods (Isopoda); however, *K. andersoni* was not found to co‐occur with the introduced *Styloniscus otakensis* , which is only present at northern sites on Macquarie Island (Houghton et al. 2022). The suppression of non‐native invertebrates by *K. andersoni* may limit their abundance and spread across Macquarie Island while enhancing the invasion of the flatworm.

The full impacts of a novel invasive invertebrate predator on ecosystem function are difficult to quantity. In addition to predation impacts, *K. andersoni* will compete with native invertebrate predators, including the spiders *Myro kerguelensis*, *Parafroneta marrineri*, and *Haplinis mundenia* , the wasp *Spilomicrus latigaster*, rove beetles, and potentially predatory mite (Acarina) species. As a scavenger of dead invertebrates and carrion (Figure 3), *K. andersoni* will also compete with native detritivores. It is important to note that native fauna may also show behavioural changes in response to invasive invertebrate predators, including altered feeding, defence, dispersal, courtship and mating, and competitive strategies as a means of avoiding predation (Ruland and Jeschke 2020). The presence of invasive predators can stimulate genetic or morphological changes within invertebrate prey populations, through selection or by decreasing prey population density (Ernsting et al. 1995; Ruland and Jeschke 2020; Zuk et al. 2006). Aggressive scavengers may also drive less competitive native fauna towards an alternate diet (e.g., greater herbivory) and lower trophic level (McNatty et al. 2009). While these more nuanced effects are not captured in our data, it is likely that *K. andersoni* will have species‐specific impacts upon invertebrate competitors and prey.

At the community scale, our findings indicate that *K. andersoni* could affect the structure and flow of energy through the food web by increasing the predation pressure across all trophic levels where it occurs (Figure 2). Within Tallgrass and *Stilbocarpa* vegetation communities, we found significant differences in invertebrate community structure. Elevation and presence of *K. andersoni* were significant drivers of invertebrate community composition. Within the surveyed sites, the highest elevation the flatworm was found was at Site 13 (189.31 m) which had the most distinct community (Figure 6) and lowest invertebrate richness (Table 1). This indicates that the impact of *K. andersoni* may be greater at high elevation sites where species richness and abundance is generally lower, as predation is more intense upon the limited taxa. At sites with greater species richness (including low elevation sites), *K. andersoni* may have a low or moderate predation impact on community structure. Several higher taxonomic groups on Macquarie Island are represented by very few native species, including Araneae (3 spp.), Coleoptera (5 spp.), Hymenoptera (1 sp.), Lepidoptera (1 sp.), Mollusca (1 sp.) and Psocoptera (1 sp.) (Greenslade and Van Klinken 2006); these may be at high risk of local extinction. This is a major concern, as loss of invertebrate groups from an ecosystem could result in loss of ecological interactions, lower functional diversity, and reduced ecosystem resilience to stress or disturbance (Audino et al. 2014; Cadotte et al. 2011; Valiente‐Banuet et al. 2015).

The rate and success of invasion is influenced by several factors, including interactions with native fauna (Ficetola et al. 2007) and biogeographic features of the ecosystem (Bazzichetto et al. 2021). The distribution of *K. andersoni* on Macquarie Island was quantified by Houghton et al. (2022), however the distribution parameters had not been explored. In this study, we identified drivers and modelled suitable habitat to determine where *K. andersoni* is likely to be found in future.

### 4.2 Distribution

Contrary to expectations, our modelling showed that flatworm distribution on Macquarie Island is driven by slope and topographically deflected wind speed. Slope and wind speed are important factors in driving invertebrate predator abundance on Macquarie Island (Houghton 2020). Slope and wind influence the flow and availability of water, which is vital for invertebrates in polar regions (Caruso et al. 2007; Day et al. 2009; Everatt et al. 2015; McGeoch et al. 2006). Wind influences soil desiccation, litter redistribution, plant growth and cover (Momberg et al. 2021; Selkirk‐Bell and Selkirk 2013) thereby affecting invertebrate habitats. Wind can also limit dispersal capabilities (Davies and Melbourne 1999), increase physiological stress in invertebrates (Chown et al. 2004; Elnitsky et al. 2008; Klok and Chown 1998), and affect hunting performance of predators (Barton 2014; Cherry and Barton 2017). Slope and wind factors therefore affect habitat suitability, physiological activity, and feeding capabilities of flatworms, while also influencing the availability and density of prey.

Topographic wetness was expected to be correlated with *K. andersoni* occurrence, as moisture is a key component of flatworm habitats (Blackshaw 1996; Boll and Leal‐Zanchet 2022; Gerlach 2019; Sugiura 2009), however this was not reflected in our models. Moisture may be partially captured with slope and wind speed. Macquarie Island maintains a consistently moist and cool climate of 3.7°C–6.6°C, with an annual precipitation of ~954 mm (Terauds et al. 2011). As a result of the frequent rainfall, moisture may not be a limiting factor; wind speed is therefore a stronger determinant of microclimate and habitat suitability for *K. andersoni* . Many west coast sites are subject to high wind exposure, with drier soils and less leaf litter (Houghton, pers. obs.); these areas are therefore less suitable as flatworm habitats. While soil moisture is vital to prevent desiccation, highly moist or flooded habitats can be unsuitable for flatworms (Carbayo et al. 2002; Froehlich and Froehlich 1972; Sluys 1999); slope may also mediate moisture levels in soil and leaf litter. It was also expected that proximity to walking tracks could infer human‐mediated dispersal (Greenslade et al. 2007; Houghton et al. 2022), however a significant correlation between *K. andersoni* occurrence and walking tracks was not found. It is important to note that human traffic across the island is not limited to walking tracks only, and could contribute to flatworm dispersal to some degree.

### 4.3 Future Habitat

Our models show there is suitable habitat and capacity for *K. andersoni* to expand its current distribution, particularly along the east coastline and north of Sellick Bay on the west coast (Figures 8 and 9). There are regions to the northwest of Green Gorge, around Mt. Law, and southwest of Lusitania Bay where additional surveys would be beneficial to determine if the flatworm has spread further inland. Much of the island plateau appears to be unsuitable habitat.

In the sub‐Antarctic many invasive invertebrates may expand their distribution to higher elevation and inland habitats, with climate change (Daly et al. 2023; Ouisse et al. 2020). Macquarie Island is predicted to continue to warm, with changes to landscape microclimates and ecosystem processes (Nel et al. 2023). *Kontikia andersoni* may expand its range to higher elevations across the island in future. This is a concern, as we show the impacts of *K. andersoni* are likely to be more severe at higher elevations.

### 4.4 Implications for Management

The management and control of invasive species on islands has improved in recent decades, however in many cases invertebrate eradication is extremely challenging, costly, or impractical (Simberloff et al. 2018). Successful invertebrate eradications have mainly occurred on small islands or limited areas within larger islands (Fowler 2003; Simberloff et al. 2018). There are instances where the long‐term suppression and control of invasive populations of invertebrates can be beneficial (Gaigher et al. 2012; Simberloff et al. 2018).

Activities in the Antarctic and sub‐Antarctic are heavily regulated to limit environmental damage. Control or eradication of invertebrates may be difficult or unachievable in these ecosystems (Bartlett et al. 2021), or may require complex strategies, multiple treatment actions, and regular monitoring (Bergstrom et al. 2018; Springer 2016). While there has been success in managing and eradicating vertebrates on Macquarie Island and other sub‐Antarctic islands (Springer 2016) there has been little success in eradication or control of invasive invertebrates in the region.

Globally, terrestrial flatworms have few predators (Ducey et al. 1999; Lemos et al. 2012; Stokes et al. 2014). There is evidence that larvae and adults of some carabid and staphylinid beetles predate flatworms (*Artiposthia triangulata*) in New Zealand (Gibson et al. 1997). Native staphylinid beetles on Macquarie Island are thought to predate fly larvae (Greenslade and Van Klinken 2006). Laboratory feeding trials are needed to assess interactions between *K. andersoni* and staphylinid beetles on Macquarie Island.

The Tasmanian fungus gnat *Planarivora insignis* is the only recorded parasitoid of flatworms (Hickman 1965). Hosts include three known Geoplanidae, but not *K. andersoni* (Hickman 1965; Winsor and Stevens 2005). While there is one fungus gnat present on Macquarie Island *(B. strenua* ), its diet consists of plant material (Broadley et al. 2018). Biocontrol agents have only been used on Macquarie Island for the control of vertebrates (Copson and Whinam 2001; Sobey et al. 1973; Springer 2016). Parasitoids can be effective invertebrate biocontrol agents (Batta 2020; Enkegaard et al. 2013; Wang et al. 2019) and as specialists, they may present little risk to native invertebrates. One such example is the wasp *Aphidius matricariae* which was accidentally introduced on Marion Island *ca*. 2001 and parasitizes the non‐native aphid *Rhopalosiphum padi* (Lee and Chown 2016). This appears to be a positive (though unintentional) application of a biocontrol agent within the sub‐Antarctic; however, biocontrol options require considerable research and assessment of management and legislative requirements. Biocontrol agents must be carefully investigated to avoid potentially devastating impacts on non‐target species (Copson and Whinam 2001; Cowie 2001; Gerlach et al. 2021; Louda et al. 2003).

Biosecurity measures within the Antarctic region currently include checking equipment and clothing, and boot washing and scrubbing with disinfectants such as the microbial biocide Virkon S, which is not effective on invertebrates (Bartlett et al. 2021). New information on life history, physiological tolerances, movement rates, and reproductive strategy would improve our understanding of *K. andersoni* (Baird et al. 2005; Justine et al. 2018; McDonald and Jones 2014) and inform effective management strategies (Justine et al. 2014; Sugiura 2010b). Some flatworms may be susceptible to heat, warm water, salt, or pesticides (such as gamma HCH) (Blackshaw 1996; Justine et al. 2014). Hot water immersion at 43°C–50°C for 5 min has resulted in 100% mortality for invasive flatworms and other soil fauna (Sugiura 2010b). This is likely to be the best method for destruction of individual flatworms or egg capsules, and for treating clothing, equipment, and cargo. Treatments may be costly or impractical to implement at a large scale but could be effective control methods at checkpoints along Macquarie Island, to prevent further spread of *K. andersoni* .

Persons travelling between sites where the flatworm is present and where it is absent (regions further north or inland from the coast) should consider specific biosecurity measures to reduce spread via boots, walking poles, or other equipment. The flatworm does not currently occur at the research station on the Isthmus or Sandy Bay, which are areas of high activity with cargo, boat, and helicopter landings, and tourist visitation. As *K. andersoni* has not been introduced to Australia, it is vital to prevent its transportation from Macquarie Island to the mainland (Greenslade et al. 2007).

## 5 Conclusion

Conservation of island ecosystems is vital to conserve global biodiversity (Courchamp et al. 2014; Horn et al. 2022; Kier et al. 2009), however extinction and biodiversity loss is pronounced on islands (Cotoras et al. 2021; Tershy et al. 2015). Terrestrial invertebrates are a critical component of island food webs and ecosystem functioning, however research has shown that invertebrate biomass is in rapid decline worldwide (Collen et al. 2012; Finn et al. 2023). Extinction rates and threat levels for invertebrates may be exceedingly underestimated (Collen et al. 2012; Finn et al. 2023) therefore the conservation of invertebrate diversity and abundance should be a priority (Hallmann et al. 2017).

Invasive species have profound detrimental effects upon island ecosystems (Fowler 2003; Houghton et al. 2019; Lebouvier et al. 2011, 2020). The impacts of invasive species (including invertebrates) in the sub‐Antarctic region are understudied (Baird et al. 2020; Convey et al. 2010). Invasion success is driven by a multitude of factors, including ecosystem biogeographic characteristics (Bazzichetto et al. 2021; Schmack et al. 2020), and biotic interactions between the invasive species and the native community (Ficetola et al. 2007). Our work investigates an invasive invertebrate predator, and its effects on the invertebrate communities of a remote oceanic island in the sub‐Antarctic. Through combined explorations of literature, examining invertebrate communities across multiple sites, and modelling, we provide valuable insights into the mechanics underlying the invasion of *K. andersoni* on Macquarie Island. We identify suitable habitats for *K. andersoni* , and regions to target for additional surveys. Our work supports the selection of pro‐active, effective biosecurity measures and management actions.

## Author Contributions

**Kita M. Williams:** conceptualization (equal), data curation (equal), formal analysis (equal), investigation (lead), methodology (equal), visualization (lead), writing – original draft (lead), writing – review and editing (lead). **Justine Shaw:** conceptualization (equal), investigation (supporting), supervision (lead), writing – original draft (supporting), writing – review and editing (supporting). **Samuel Waite:** conceptualization (supporting), data curation (equal), formal analysis (equal), investigation (supporting), methodology (equal), software (supporting), visualization (supporting), writing – original draft (supporting), writing – review and editing (supporting). **Melissa Houghton:** data curation (equal), investigation (supporting), methodology (equal), visualization (supporting), writing – review and editing (supporting). **Jennifer Firn:** formal analysis (supporting), supervision (supporting), writing – review and editing (supporting).

## Disclosure

The authors have nothing to report.

## Conflicts of Interest

The authors declare no conflicts of interest.

## Supporting information


Data S1.

Data S2.

Data S3.

Data S4.

Data S5.

Data S6.

## Data Availability

The authors confirm that the data supporting the findings of this study are available within the article and its Supporting Information—S1. Data from 2015 to 2018 island‐wide invertebrate trapping program is available at: doi: 10.26179/5ba86d73e923c

## Appendix A

**TABLE A1:** Invertebrate trapping sites on Macquarie Island (2015–18).

Site	Site location	Latitude	Longitude	Elevation (m)	Vegetation
Site 1	Wireless Hill	−54.49389	158.94165	92.88	Shortgrass
Site 2	Wireless Hill	−54.49384	158.94148	93.06	Tallgrass
Site 3	Gadget's Gully	54.494666	158.942586	166.2	Feldmark
Site 4	Wireless Hill	−54.494666	158.942586	89.99	Herbfield
Site 5	Halfway Hill	−54.51439	158.93379	45.58	Shortgrass
Site 6	Razorback	−54.506	158.93256	7.39	Stilbocarpa
Site 7	Razorback	−54.50422	158.9328	7.44	Tallgrass
Site 8	Doctor's Track	−54.50626	158.92586	162.41	Tallgrass
Site 9	ILT North of Scoble Lake	−54.51475	158.91508	242.22	Feldmark
Site 10	ILT North of Scoble Lake	−54.51493	158.91412	235.64	Herbfield
Site 11	Green Gorge	−54.62707	158.89618	24.56	Shortgrass
Site 12	Green Gorge	−54.63174	15,889,864	20	Stilbocarpa
Site 13	Rockhopper Bay Escarpment Edge	−54.69127	158.81758	189.31	Tallgrass
Site 14	Tiobunga Lake	−54.6954	158.82217	197.61	Feldmark
Site 15	West of Overland, South of Whiskey Creek	−54.75795	158.82498	236.07	Herbfield
Site 16	Links Track	−54.76844	158.82072	301.96	Feldmark
Site 17	Hurd Point	−54.77204	158.82507	15.45	Tallgrass
Site 18	Waterfall Bay	−54.68934	158.86383	35.14	Stilbocarpa
Site 19	Bauer Bay	−54.55576	158.8765	30.24	Stilbocarpa
Site 20	Bauer Bay	−54.55151	158.87831	44.14	Herbfield
Site 21	Handspike	−54.50089	158.89096	39.61	Tallgrass
Site 22	Handspike	−54.50003	158.89137	25.66	Shortgrass
Site 23	Sandell Bay	−54.67619	158.83072	12.32	Tallgrass
Site 24	Lusitania Bay	−54.71928	158.84929	29.32	Stilbocarpa

**TABLE A2:** Flatworm predation literature, including the prey and predation behavior of genus *Kontikia* and other flatworm genera.

Flatworm prey/predation behaviour	Flatworm genus	References
Gastropoda (Slugs)	*Kontikia*	Winsor and Stevens 2005
Gregarious attacks on prey	*Kontikia*	Winsor and Stevens 2005
Acarina (Mites)	*Geoplana*	Froehlich 1955
	*Xerapoa*	Cseh et al. 2017
	Genus not specified	Lemos et al. 2012
Annelida (Earthworms)	*Arthurdendyus*	Murchie and Gordon 2013
	*Artioposthia*	Gibson et al. 1997; Haria 1995
	*Bipalium*	Fiore et al. 2004; Ogren 1995
	*Dolichoplana*	Ogren 1995
	*Geobia*	Cseh et al. 2017; Ogren 1995
	*Geoplana*	Cseh et al. 2017; Barker 1989
	*Imbira*	Cseh et al. 2017; Cuevas‐Caballé et al. 2019
	*Microplana*	McDonald and Jones 2014; Ogren 1995
	*Obama*	Boll and Leal‐Zanchet 2016; Roy et al. 2022
	*Platydemus*	Ohbayashi et al. 2005; Sugiura 2010a
	*Rhynchodemus*	Ogren 1995
Arachnida (Spiders and Harvestman)	*Cephaloflexa*	Cseh et al. 2017; Cuevas‐Caballé et al. 2019; Silva et al. 2018
	*Caenoplana*	Jones et al. 2020
	*Choeradoplana*	Cardoso et al. 2023; Cseh et al. 2017
	*Geoplana*	Froehlich 1955
	*Xerapoa*	Cseh et al. 2017
Collembola (Springtails)	*Geoplana*	Froehlich 1955
	*Microplana*	Jennings 1959
	*Rhynchodemus*	Froehlich 1955; Ogren 1995
	Genus not specified	Nakamori and Suzuki 2012; Sluys 1999; Sluys 2019; Winsor and Stevens 2005
Gastropoda (Slugs)	*Bipalium*	Ogren 1995
	*Endeavouria*	Boll et al. 2015
	*Geoplana*	Barker 1989; Cseh et al. 2017
	*Microplana*	McDonald and Jones 2014
	*Obama*	Boll and Leal‐Zanchet 2016; Boll et al. 2020; Cseh et al. 2017
	*Paraba*	Boll and Leal‐Zanchet 2016
	*Parakontikia*	Ogren 1995
	*Platydemus*	Gerlach 2019; Ohbayashi et al. 2005
	*Rhynchodemus*	Ogren 1995
Gastropoda (Snails)	*Bipalium*	Ogren 1995
	*Endeavouria*	Boll et al. 2015; Ogren 1995
	*Geoplana*	Barker 1989; Cseh et al. 2017
	*Obama*	Boll and Leal‐Zanchet 2016; Cseh et al. 2017
	*Paraba*	Boll and Leal‐Zanchet 2016
	*Platydemus*	Gerlach 2019; Iwai et al. 2010; Ohbayashi et al. 2005; Sugiura 2009; Sugiura and Yamaura 2009
	*Rhynchodemus*	Ogren 1995
Hymenoptera (Ants, Bees, Wasps)	*Cephaloflexa*	Cuevas‐Caballé et al. 2019
Isopoda (Woodlice)	*Caenoplana*	Jones et al. 2020; Ogren 1995
	*Cephaloflexa*	Cseh et al. 2017; Cuevas‐Caballé et al. 2019
	*Endeavouria*	Boll et al. 2015
	*Geoplana*	Barker 1989; Cseh et al. 2017; Froehlich 1955
	*Issoca*	Cseh et al. 2017
	*Luteostriata*	Boll and Leal‐Zanchet 2016, 2018; Cseh et al. 2017
	*Microplana*	McDonald and Jones 2014
	*Notogynaphallia*	Prasniski and Leal‐Zanchet 2009
	*Parakontikia*	Barker 1989; Ogren 1995
	*Pasipha*	Cseh et al. 2017
	*Platydemus*	Sugiura 2010a
	*Xerapoa*	Cseh et al. 2017
Insecta (Insects and insect larvae)	*Caenoplana*	Jones et al. 2020; Ogren 1995
	*Cephaloflexa*	Cseh et al. 2017; Cuevas‐Caballé et al. 2019
	*Geoplana*	Barker 1989; Froehlich 1955
	*Notogynaphallia*	Cseh et al. 2017
	*Matuxia*	Ogren 1995
	*Microplana*	Jones et al. 1990; Ogren 1995
	*Notogynaphallia*	Cseh et al. 2017
	*Rhynchodemus*	Ogren 1995
Platyhelminthes (Flatworms)	*Geoplana*	Cseh et al. 2017; Froehlich 1955
	*Notogynaphallia*	Cseh et al. 2017
	*Obama*	Boll et al. 2015; Boll et al. 2020; Boll and Leal‐Zanchet 2016, 2018; Cseh et al. 2017
	*Paraba*	Boll et al. 2015; Boll and Leal‐Zanchet 2016
	*Platydemus*	Ohbayashi et al. 2005
Gregarious attacks on prey	*Endeavouria*	Boll et al. 2015
	*Geoplana*	Barker 1989
	*Imbira*	Cseh et al. 2017
	*Parakontikia*	Ogren 1995
	*Platydemus*	Ohbayashi et al. 2005; Sugiura 2010a
Scavenging of dead invertebrates	*Endeavouria*	Boll et al. 2015; Ogren 1995
	*Geoplana*	Barker 1989
	*Microplana*	McDonald and Jones 2014
	*Platydemus*	Gerlach 2019; Ohbayashi et al. 2005

**TABLE A3:** Environmental spatial layers used to model the distribution and habitat suitability of *K. andersoni* .

Variable/layer	Description	Ecological relevance	Source/methods	Spatial resolution	Units or categories
*Terrain/environmental*					
Aspect	Compass direction that a terrain surface faces	Influences exposure to solar radiation, and alters precipitation and evapotranspiration frequency (Yang et al. 2020)	5 m resolution DEM using GDAL (Geospatial Data Abstraction Library) (Bricher et al. 2013)	5 m	Degrees (°)
Elevation	Distance above sea level of a terrain surface	Increasing elevation is linked to increases in wind exposure, probability of fog, and relative humidity, and decreases in air temperature and soil temperature (Davies and Melbourne 1999; Fitzgerald et al. 2022). Plant and invertebrate species distributions are often driven by elevation gradient (Davies and Melbourne 1999; Terauds et al. 2011)	5 m resolution DEM of Macquarie Island derived from Airborne Synthetic Aperture RADAR data acquired in 2000 by the NASA PACRIM Mission 2 (Bricher et al. 2013)	5 m	Metres (m)
Slope	Steepness/gradient/inclination of a terrain surface	Increasing slope increases drainage rate, and frequency of stochastic disturbance (Scott and Kirkpatrick 2008)	5 m resolution DEM using GDAL (Geospatial Data Abstraction Library) (Bricher et al. 2013)	5 m	Degrees (°)
Topographic Wetness Index	An index that identifies areas for potential water accumulation or adequate drainage. Calculated on a per pixel base using the following formula: $\ln\left(\frac{\text{Specific Cathcment Area}}{\tan\left(\text{slope angle}\right)}\right)$	Indicator of waterlogging or drainage within a specific area, and a surrogate for soil depth and level of organic matter (Fitzgerald et al. 2022). Moisture is correlated with invertebrate species richness (McGeoch et al. 2006; Terauds et al. 2011)	5 m resolution DEM using SAGA (System for Automated Geoscientific Analyses) GIS (Bricher et al. 2013)	5 m	Unitless
Topographically Deflected Wind Speed	Model estimating wind speed across Macquarie Island. This factors in prevailing wind from a west and north‐west direction, as well as a mean wind speed of 35.1 km/h	Increased wind exposure and windspeed can cause disturbance and soil desiccation, re‐distribute litter, and limit plant growth and vegetation cover (Momberg et al. 2021; Selkirk‐Bell and Selkirk 2013)	5 m resolution DEM using GDAL (Geospatial Data Abstraction Library) (Bricher et al. 2013)	5 m	Kilometres per hour (km/h)
*Disturbance*					
Proximity to Seal or Penguin Colonies	Distance from known penguin and seal colony locations	Colonies are sources of natural disturbance (Haussmann et al. 2013)	Proximity Raster created using SAGA Next Gen GIS and a combined seal and penguin colony polygon layer (Harris 2015a)	5 m	Metres (m)
Proximity to Walking Tracks	Distance from established walking tracks	Walking tracks are known pathways for anthropogenic‐mediated dispersal and a source of disturbance (Shaw 2013).	Proximity Raster created using SAGA Next Gen GIS and walking track polyline layer (Harris 2015b)	5 m	Metres (m)
Proximity to Watercourses and Lakes	Distance from known watercourses and lakes	Water availability is a key limiting factor in the abundance and distribution of flora and fauna in the Antarctic region (Kennedy 1993)	Proximity Raster created using SAGA Next Gen GIS, Water Course polyline and lake polygon layers (Brolsma 2012)	5 m	Metres (m)
