# Supplementary material for: A Novel Invertebrate Predator on an Oceanic Island: Impacts and Invasion Dynamics of Kontikia andersoni on Macquarie Island
Source: Ecol Evol. 2025 Jul 1;15(7):e71663. doi: 10.1002/ece3.71663 (PMC12209868; doi:10.1002/ece3.71663)
Supplement: Supplementary file 6 — Data S6. [file ECE3-15-e71663-s001.docx]

**Supplementary Information**

**Habitat suitability modelling**

Spatial autocorrelation within the model residuals was tested for using Moran’s I test (Moran, 1950) and a Mantel correlogram (Mantel, 1967) via the *ape* (Paradis and Schliep, 2019) and *ncf* (Bjornstad, 2022) packages respectively. Significant spatial autocorrelation was found within the residuals (*p*-value = 0.04).

Model term selection to identify the most parsimonious model was then performed automatically using the ‘shrinkage approach’ (Marra and Wood, 2011), in which each model term is smoothed using the ‘shrinkage’ version of the thin plate regression spline. This process penalises non-important smooth terms to the zero function and by way selects them ‘out’ of the model (Marra and Wood, 2011), as well as preventing the need to perform traditional model selection processes (i.e., forwards or backwards selection). All model variables that shrunk to zero and were non-significant, as assessed by the model summary and variable plots, were subsequently dropped and each model was refitted using the remaining terms. A spatial bivariate smooth (site easting and northing (UTM 57S)) was included to account for spatial autocorrelation within the model residuals.

**Hyperparameter optimisation for Boosted Regression Trees (BRT), Random Forest (RF), Artificial Neural Networks (ANN), and Extreme Gradient Boosting (XGBoost) modelling**

**SI TABLE 1 |** Hyperparameter optimisation for Boosted Regression tree modelling to predict island-wide habitat suitability for *K. andersoni*. The range of values for each hyperparameter and the optimal combination of parameters for each model type are listed, with optimal parameters for both training iterations (initial and final) presented.

| Hyperparameter | Description | Range of Values Assessed | Optimal Value  (Initial Selection) | Optimal Value  (Final Selection) |
| --- | --- | --- | --- | --- |
| *n.trees* | Number of boosting iterations or individual trees within the overall model | 400, 600, 800, 1000, 1200, 1400 | 1000 | 1200 |
| *interaction.depth* | Maximum tree depth/complexity – the number of leaf nodes within a tree | 2,3,5,7,9 | 2 | 5 |
| *shrinkage* | shrinkage/learning rate – controls the contribution of each tree as its added to the overall model | 0.01, 0.005, 0.001, 0.0005 | 0.01 | 0.01 |
| *n.minobsinnode* | Minimal number of samples required to be at a leaf node | 3,5,7,9 | 9 | 5 |
| *Bag.fraction* | Bag fraction – specifies the proportion of the data to be drawn at random, without replacement, from the full training set for each step/tree | 0.75 | 0.75 | 0.75 |
| *Distribution Type* | The distribution type selected for the model | ‘bernoulli’ , | ‘bernoulli’ | ‘bernoulli’ |

**SI TABLE 2 |** Hyperparameter optimisation for Random Forest modelling to predict island-wide habitat suitability for *K. andersoni*. The range of values for each hyperparameter and the optimal combination of parameters for each model type are listed.

| Hyperparameter | Description | Range/Number of Levels Assessed | Optimal Value |
| --- | --- | --- | --- |
| mtry | Number of randomly selected predictors to be considered for each tree | 2, 3, 4, 5, 6 | 2 |
| *splitrule* | Rule by which each split is considered in a tree | "gini", "extratrees","hellinger", | extratrees |
| *min.node.size* | Minimal number of samples required to be at a leaf node | 1, 3, 5, 7, 9 | 9 |

**SI TABLE 3 |** Hyperparameter optimisation for Artificial Neural Network modelling to predict the island-wide habitat suitability of *K. andersoni.* The range of values for each hyperparameter and the optimal combination of parameters for each model type are listed.

| Hyperparameter | Description | Range/Number of Levels Assessed | Optimal Value |
| --- | --- | --- | --- |
| size | number of units in the hidden layer of the neural network | 2, 3, 4, 5, 6 | 2 |
| *decay* | parameter for weight decay, designed to regulize the weights | 0.1, 0.05, 0.01, 0.005, 0.001, 0.0005 | 0.01 |

**SI TABLE 4 |** Hyperparameter optimisation for Extreme Gradient Boosting modelling to predict the island-wide habitat suitability of *K. andersoni.* The range of values for each hyperparameter and the optimal combination of parameters for each model type are listed, with optimal parameters for both training iterations (initial and final) presented.

| Hyperparameter | Description | Range/Number of Levels Assessed | Optimal Value  (Initial Selection) | Optimal Value  (Final Selection) |
| --- | --- | --- | --- | --- |
| nrounds |  | 100, 150, 200, 250, 300 | 100 | 100 |
| *lambda* |  | 0.1, 0.18, 0.32, 0.56, 1.0 | 0.56 | 1 |
| alpha |  | 1 | 1 | 1 |
| *eta* |  | 0.1, 0.2, 0.3, 0.4, 0.5 | 0.1 | 0.1 |

**Model Selection for the predictive modelling of *K. andersoni* occupancy on Macquarie Island**

**SI TABLE 5 |** Initial model selection for the predictive modelling of *K. andersoni* occupancy on Macquarie Island. Generalised Additive Models (GAM), Boosted Regression Trees (BRT), Random Forests (RF), Artificial Neural Networks (ANN) and Extreme Gradient Boosting (XGBoost) were trialled and evaluated using 10-fold block cross-validation on training data. The mean Cohen’s Kappa Statistic (Cohen, 1960) value across the 10 folds was used to assess performance.

| Model Type | Mean Kappa | |
| --- | --- | --- |
| GAM | | 0.24 |
| BRT | | 0.42 |
| RF | | 0.35 |
| ANN | | 0.36 |
| XGBoost | | 0.44 |

**SI TABLE 6 |** Final model selection for the predictive modelling of *K. andersoni* occupancy on Macquarie Island. Boosted Regression Trees (BRT) and Extreme Gradient Boosting (XGBoost) were trialled and evaluated without Topographic Wetness Index and Proximity to Walking Tracks using 10-fold block cross-validation on training data. The mean Cohen’s Kappa Statistic (Cohen, 1960) value across the 10 folds was used to assess performance.

| Model Type | Mean Kappa | |
| --- | --- | --- |
| GAM | | 0.53 |
| XGBoost | | 0.41 |

**References**

Bjornstad, O. N. (2022). *ncf: Spatial Covariance Functions*. https://CRAN.R-project.org/package=ncf

Cohen, J. (1960) A Coefficient of Agreement for Nominal Scales*. Educational and Psychological Measurement*, *20*(1), 37-46. <http://dx.doi.org/10.1177/001316446002000104>

Mantel, N. (1967). The detection of disease clustering and a generalized regression approach. *Cancer Research*, *27*(2), 209–220.

Marra, G., Wood, S. N. (2011). Practical variable selection for generalized additive models. *Computational Statistics & Data Analysis*, *55*(7), 2372–2387.

Moran, P. A. P. (1950). Notes on continuous stochastic phenomena. *Biometrika*, *37*(1–2), 17–23.

Paradis, E., Schliep, K. (2019). ape 5.0: an environment for modern phylogenetics and evolutionary analyses in R. In *Bioinformatics*: *35*, 526-528. https://doi.org/10.1093/bioinformatics/bty633
